# Inactivation of *Rhizoctonia solani* in fertigation water using regenerative *in situ* electrochemical hypochlorination

**DOI:** 10.1038/s41598-019-50600-7

**Published:** 2019-10-02

**Authors:** Serge Lévesque, Thomas Graham, Dorin Bejan, Jamie Lawson, Ping Zhang, Mike Dixon

**Affiliations:** 1 Controlled Environment Systems Research Facility, School of Environmental Science, University of Guelph, Guelph, Ontario N1G 2W1 Canada; 2 Environmental Technology Consultant for CESRF, 275 Royalton Common Unit 49, Oakville, Ontario L6H 0N2 Canada

**Keywords:** Plant sciences, Environmental chemistry

## Abstract

The capture and re-use of greenhouse fertigation water is an efficient use of fertilizer and limited water resources, although the practice is not without risk. Plant pathogens and chemical contaminants can build up over successive capture and re-use cycles; if not properly managed they can lead to reduced productivity or crop loss. There are numerous established and emerging water treatment technologies available to treat fertigation water. Electrochemical processes are emerging as effective means for controlling pathogens via *in situ* regenerative hypochlorination; a process that is demonstrated here to achieve pathogen control in fertigation solutions without leading to the accumulation of potentially phytotoxic free chlorine residuals associated with other chlorination processes. An electrochemical flow cell (EFC) outfitted with ruthenium dioxide (RuO_2_) dimensionally stable anodes (DSA) was characterized and evaluated for free chlorine production and *Rhizoctonia solani* inactivation in both irrigation and fertigation solutions. Pathogen inactivation was achieved at low current densities and short residence or cell contact times. Effluent free chlorine concentrations were significantly lower than commonly reported phytotoxic threshold values (approximately 2.5 mg/L) when fertilizer (containing ammonium) was present in the test solution; an effect attributable to reactions associated with breakpoint chlorination, including chloramine formation, as well as the presence of other oxidizable compounds in the fertilizer. Chloride concentrations were stable under the test conditions suggesting that the EFC was operating as a regenerative *in situ* electrochemical hypochlorination system. No significant changes to macronutrient concentrations were found following passage through the EFC.

## Introduction

Greenhouse production, by its very nature, is reliant on irrigation for crop production. In nearly every major greenhouse production region the demands on local water resources are substantial, often resulting in scarcity^1^. In addition to the limits on availability, there are also growing concerns over the quality of those water resources. Human activities, including intensive agricultural and industrial practices, have led to degraded source waters that may no longer be suitable for irrigation^2–4^. Irrigation source waters can contain a wide range of chemical contaminants including pesticides, herbicides, growth regulators, plasticizers, and pharmaceuticals, as well as biological contaminants (*Phytophthora spp*., *Pythium spp*., *Rhizoctonia spp*., *Fusarium oxysporum*, *Erwinia spp*. & *Xanthomonas spp*.)^5–9^, all of which can negatively impact crop production and increase the number of unsaleable plants^1^.

Diminishing supplies and increasing costs for water are driving a shift towards the capture and reuse of irrigation/fertigation water^1,10,11^. There are numerous environmental and economic advantages to recirculating greenhouse irrigation water including improved water use efficiency, and reduced fertilizer costs^12,13^. Although an effective resource conservation tool, there are significant challenges associated with capture and re-use irrigation systems including increased risk of pathogen proliferation, the potential for accumulation of [potentially] phytotoxic chemical contaminants and nutrient imbalances in irrigation systems^12,14–16^. There are numerous treatment technologies available to growers, many of which have been reviewed by Stewart-Wade (2011) and others^8,17,18^.

Chlorination is a widely used water treatment technology, or more precisely, a group of closely related technologies. Although well established and effective at inactivating microorganisms, there are some significant drawbacks associated with chlorination of irrigation water. The continuous addition of free chlorine, including hypochlorous acid (HOCl), hypochlorite (ClO^−^), or injection of chlorine gas (Cl_2(g)_) in water will result in a variable distribution and stability of free chlorine species that have differing pathogen inactivation efficacies depending on specific solution conditions (e.g., pH). Ultimately this variability will affect the contact times necessary for pathogen inactivation^19^. Some pathogens, such as *Fusarium oxysporum* and *Rhizoctonia solani*, require a residual free chlorine concentration of 8–14 mg/L with contact times on the order of 5–10 minutes to achieve inactivation^19–21^. The reported free chlorine levels required to control the spread of these and other plant pathogens is greater than the commonly reported free chlorine phytotoxic threshold of ~2.5 mg/L for many crop species^18,20,22,23^. Further, continual addition or injection of free chlorine can lead to the accumulation of chloride that if left unchecked can lead to crop damage/loss^24^. In standard chlorine injection systems residual free chlorine and chloride levels must be continuously monitored to ensure phytotoxic levels are not reached. In addition to free chlorine and chloride, one must also consider chlorine reaction products that could also affect production. When using free chlorine, and more specifically HOCl, in a hydroponic system (e.g., deep water system; nutrient film technique, etc.,) with significant amounts of ammonium-based nitrogen sources, chloramine species, specifically NH_2_Cl, can form. The combination of free chlorine and chloramines can result in lower phytotoxicity thresholds for the solution as a whole (e.g., 0.3 mg/L HOCl and 0.2 mg/L NH_2_Cl)^25,26^. This can further complicate irrigation solution management requirements for growers. Finally, there are also worker safety concerns when handling chlorine disinfectants, especially chlorine gas, which can cause pulmonary health effects at low levels^20,21^.

Chlorination of irrigation water could also be achieved via electrochemical processes that, if properly managed, could avoid many of the limitations of traditional chlorination procedures^27,28^. Electrochemical chlorination dynamics are governed primarily by the composition of the electrodes, the applied current, and the chemical composition of the solution^29,30^. The nature of the electrode material governs the types of anodic and cathodic reactions that can occur, including direct and mediated electrolysis, which could contribute to microbial inactivation^31^. Electrode materials that have demonstrated utility in microbial inactivation include ruthenium and iridium-based oxides, platinum, carbon (graphite), ceramics (Ti_4_O_7_) and boron-doped diamond (BDD)^32–35^. One such class of electrodes are dimensionally stable anodes (DSA) based on Ruthenium (IV) oxide (RuO_2_), which are widely used for commercial production of chlorine and chlorine oxides^34,35^.

**Figure 1:**
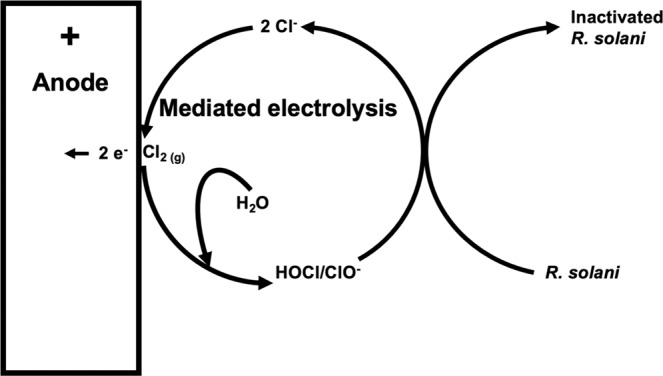
Schematic showing proposed mechanisms of pathogen inactivation with an emphasis on the cycle of reactions constituting regenerative *in situ* electrochemical hypochlorination (mediated electrolysis). Direct oxidation and other unknown inactivation mechanisms (e.g., Electroporation, high acidity near the electrode surface and/or production of hydrogen peroxide) require additional research to characterize. Regenerative mediated electrolysis, in which chloride ions are transformed to free chlorine forms (e.g., Hypochlorous acid), neutralizes pathogens and in the process releases chloride ions back to the solution. The chloride ion migrates back to the anode through electrostatic migration where it once again can be transformed to free chlorine forms leading to further pathogen inactivation.

*In situ* regenerative electrochemical hypochlorination works through mediated electrolysis, which can be utilized to inactivate pathogens and degrade pollutants in solution by regenerating free chlorine from chloride ions released during reactions with contaminants (Fig. 1)^35,36^. The continuous formation of free chlorine and recycling of chloride ions in relation to the chlorine demand can be used to inactivate and/or degrade pathogens and chemical pollutants^36–38^.

The regeneration of chloride ions occurs as free chlorine reacts with components of cell membranes, enzymes, proteins, and nucleic acids of microorganisms^39^. Hypochlorous acid (HOCl), which predominates at neutral pH, is a more effective form of free chlorine than hypochlorite (OCl^−^), which predominates at high pH. Hypochlorous acid, unlike the negatively charged OCl^−^ form of free chlorine, is neutral. Being neutral allows HOCl to more readily pass through cell membranes where it can react with a wide range of cellular components^39^. The chlorine in HOCl is electrophilic and readily reacts with molecules having high electron densities. These oxidative reactions interfere with cellular processes and membrane integrity, which ultimately leads to metabolic disruption, increased permeability of the cell, and eventually cell death^39–41^. Upon reacting, free chlorine is often reduced back to chloride (Cl^−^)^41^, at which point it can migrate back to the anode to once again be transformed into HOCl (Fig. 1). Exerting control over the various factors that regulate the regeneration of free chlorine could allow an operator to achieve effective pathogen inactivation while managing residual free chlorine to avoid phytotoxicity.

The current study was conducted to determine: (1) if RuO_2_ DSAs could be used, *in situ*, to generate sufficient free chlorine to achieve inactivation of *Rhizoctonia solani* in a fertigation solution, where the background chloride concentrations are compatible with hydroponic crop production (i.e., below phytotoxic thresholds), and (2) if the free chlorine produced *in situ* reached phytotoxic thresholds as reported in the literature.

## Materials and Methods

### Pathogen cultures

A pure culture of *Rhizoctonia solani* was provided courtesy of Dr. Allen Xue from the Plant Pathology Ottawa Research and Development Centre, Agriculture and Agri-Food Canada. Mycelia were inoculated onto potato dextrose agar (PDA) (B213400, Fisher Scientific, Canada) containing 0.1 g/L of streptomycin sulfate (BP910-50, Fisher Scientific, Canada) and 0.05 g/L of ampicillin sodium salt (BP1760-25, Fisher Scientific, Canada). The plates were then incubated on the laboratory bench inside a clear plastic container at room temperature (∼ 23 °C) and ambient light levels. After incubation for seven days the plates were fully covered with mycelia, at which point five 1 cm^2^ sections of the mycelia mat were excised from the outer edge of the petri dish and placed into 250 mL Erlenmeyer flasks, each containing 100 ml Potato Dextrose Broth (PDB) (B254920, Fisher Scientific, Canada). The suspension was then placed in an incubator (Innova 4340, New Brunswick Scientific, USA) for 8 days at 30 °C under a 12 h photoperiod. The cultured mycelia were then transferred to a blender (HH-362, E.F. Appliances Canada LTD.) and blended for 30 seconds. A pipette (4642110, Thermo Scientific, USA) was used to inoculate 10 ml of the suspension into new Erlenmeyer flasks with 250 mL of PDB and further incubated for another 8 days under the same conditions previously described.

### Test solution preparation

*Rhizoctonia solani* cultures were separated by vacuum filtration through a 1.5 μm filter disk (Whatman 934 – AH) to separate the mycelia from the liquid broth. The mycelia were rinsed off the filter disk with deionized (DI) water and deposited in a 500 mL sterile beaker and filled to 300 mL with DI water. The suspension was then transferred to a blender and blended for 1 minute. The blended suspension was then added to a 60-litre reservoir containing 30 litres of DI water. This solution was then subjected to the electrochemical treatment.

The fertilizer solutions with added chloride, in the form of potassium chloride ((P330-500), Fisher Scientific, Canada), were prepared by weighing out (TE 124 S, Sartorius, Germany) appropriate amounts of stock material (20-8-20 Plant Prod, 10561, Master Plant-Prod Inc., Canada; ammonium sulphate (A702-3) and potassium chloride) to bring the final solution volume to the targeted concentrations for each experiment (Table 1). The Plant Prod fertilizer consisted of Nitrate (4.3 mmol/L), Phosphate (0.55 mmol/L), Ammonium (2.64 mmol/L) and Potassium (2.80 mmol/L) at 0.5 grams per liter of solution. Other ions in trace amounts were Sulphate (40 μmol/L), Sodium (150 μmol/L), Magnesium (60 μmol/L), Calcium (30 μmol/L), chelated Iron (7 μmol/L), Manganese (3.50 μmol/L), Zinc (3.80 μmol/L), Copper (3.1 μmol/L), Boron (9.25 μmol/L), Molybdenum (0.78 μmol/L) and Nitrite was not initially present. Individual macronutrient ions from a 0.5 g/L solution were measured with a Shimadzu HPLC system consisting of a DGU-20A3 degasser, a SIL-10AP autosampler, two LC-20AT pumps, two CDD-10A VP conductivity detectors, CTO-20AC column oven, and CBM-20A system controller. Total Nitrogen was measured using a Shimadzu TNM-1 unit (Shimadzu Scientific Instruments, USA).

**Table 1:** Fertigation solution composition summary for each experiment presented.

Figure	Chloride (Cl^−^) concentration (mg/L)	Fertilizer type	Fertilizer concentration (g/L)
Fig. 3	0	N/A	0
Fig. 4a,b	20	N/A	0
Fig. 5	20	Plant Prod (20-8-20)	0 – 0.5
Fig. 6a,b	20–50	Plant Prod (20-8-20)	0.5
Fig. 7a,b	20	Plant Prod (20-8-20)	0.5
Fig. 8a,b	20	(NH_4_)_2_SO_4_	0.02

Solution compositions are cross referenced with their respective figures for clarity.

### Electrochemical flow cell and operation procedures

**Figure 2:**
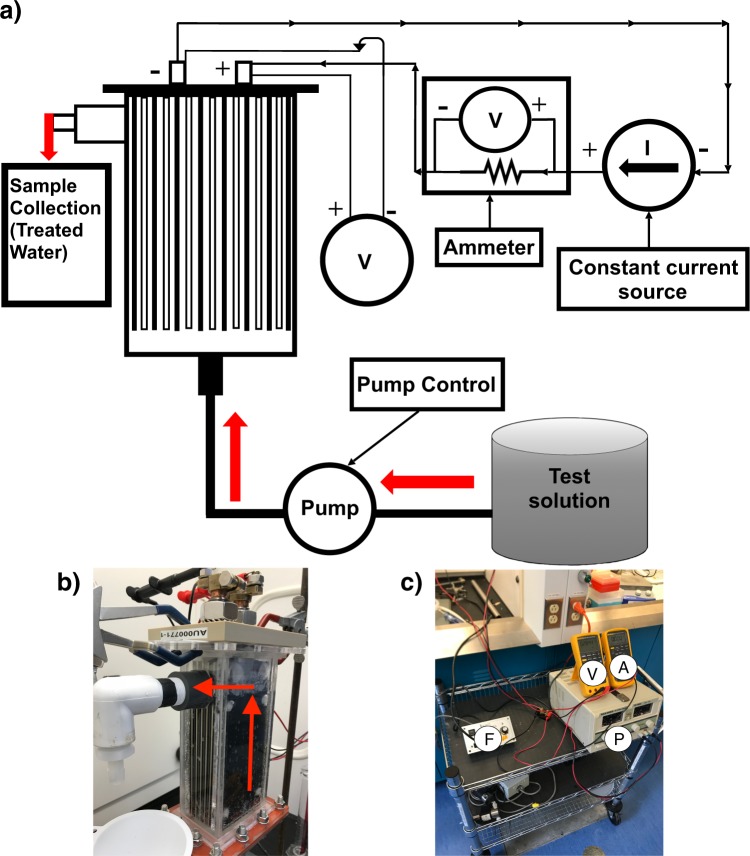
Overview of the Electrochemical Flow Cell (EFC) and supporting hardware. (**a**) Electrochemical Flow Cell (EFC) testbed schematic showing the principle components and the direction of the electrical current depicted by arrows used for the experimental setup and (**b**) the EFC with red arrows depicting the flow path and (**c**) monitoring and controlling components for flow rate (F), current (A), voltage (V), and the use of the power supply (P).

The electrochemical flow cell (EFC) system used consisted of a set of six RuO_2_ dimensionally stable anodes (DSA) (De Nora Tech, Concord, USA) and a complementary set of five stainless steel cathodes, spaced 2 mm apart, in an acrylic casing. The total area of the anodes was 1320 cm^2^. Solutions entered the cell from the bottom, passed upwards through the electrodes, and exited the cell at an outflow port at the top of the housing (Fig. 2a,b). A power supply (DF1730SC 20 A DC power supply, Gold Source, China) was connected to the anode and cathode of the flow cell. The applied current value was derived from the voltage drop measured across a precision current resistor (RS-50-100, RIEDON, Alhambra, CA) using a multimeter (Fluke 189, Fluke Corporation, Canada). A second multimeter (Fluke 87, Fluke Corporation, Canada) connected directly to the anode and cathode of the cell measured the applied voltage (Fig. 2c). A second multimeter is connected directly to the terminals of the anode and cathode array to measure the applied voltage. A variable speed peristaltic pump (Cole-Parmer 1–100 RPM) drew water from the test solution reservoir to the entry port located at the base of the flow cell. The total free volume of the EFC, the internal volume of the housing less the volume of the electrode assembly, was 380 mL. The targeted contact times were achieved by adjusting the flow rates through the cell such that a given volume of solution would remain in contact with the electrodes for the desired time interval.

Untreated samples were collected from the main reservoir at the start, middle, and end of each experiment. Colony counts from each time point were averaged and used as the control or starting value for each test condition examined in a given experiment. Three effluent samples were collected from the outlet of the EFC for each treatment combination. The first sample was collected after three cell volumes (1140 ml) had passed through the flow cell, while samples two and three were collected after one and two additional flow cell volumes had passed through the system. Samples were collected in 40 ml clear plastic HDPE vials (20120121, Richard’s Plastics, Canada). The pH, temperature (542, Corning, USA) and free chlorine concentration, for solutions containing chloride, were measured prior to microbial enumeration. Free chlorine and total chlorine from the bulk solution was measured according to the manufacturer’s methods using DPD Test ‘N Tube cuvette with free chlorine reagent set (2105545, Hach Company, USA) and total chlorine set (2503025, Hach Company, USA), and a DR/850 portable colorimeter (4845000, Hach Company, USA). Combined chlorine measurements were determined by subtracting the concentration free chlorine by that of total chlorine for each sample collected. The samples were then serially diluted (10^1^, 10^2^, 10^3^ and 10^4^) in glass test tubes and a 100 μL aliquot from each was spread onto plates with PDA and antibiotics under a laminar flow hood. Plates were inverted and incubated at 30 °C under 12-hr photoperiod at 100 μmol·m^−2^·s^−1^ photosynthetically active radiation light for 2 days. Total colony forming units per milliliter (CFU/ml) were counted after the incubation period elapsed.

### Statistical analysis

Statistical analyses were performed using JMP version 14.0 (SAS Institute Inc, Cary, NC). The residuals between data points and predicted values were tested for normality using the Shapiro-Wilk test. Data that did not pass were transformed and the analysis was conducted on the transformed data. Simple and multiple linear regression was performed on each individual experiment sets. Summary of fit from linear regression models used the adjusted R squared values and significance was determined using α = 0.05. Linear regression analysis was conducted to determine any effects on individual macronutrient ions from the fertilizer composition.

## Results & Discussion

### Control experiments

#### Zero current control

A control experiment was conducted at several contact times with solutions containing *R*. *solani* (AG-8) isolated from wheat, fertilizer (0.5 g/L Plant Prod), and 20 mg/L of chloride but without any applied current. There was no reduction in pathogen counts, indicating that there was no physical mechanism (e.g., a filtering effect) that may have been acting to reduce pathogen counts.

#### Pathogen inactivation without the presence of fertilizer or chloride

**Figure 3:**
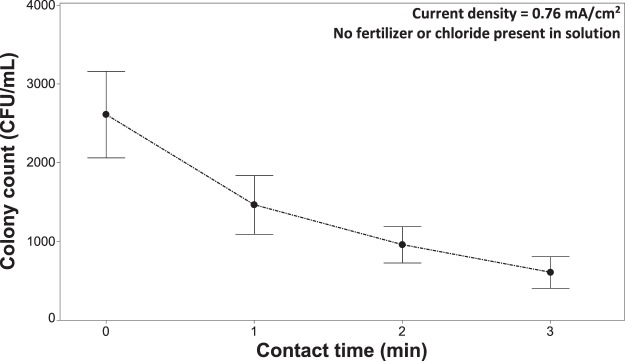
Inactivation of *Rhizoctonia solani* as a function of contact time, while maintaining a constant current density of 0.76 mA/cm^2^. The test solution only contained deionized water with free floating mycelia and did not contain fertilizer or chloride salts for the experiment. Without the presence of a supporting electrolyte in solution gave an operating voltage for this specific experiment ∼32 volts. Error bars are ±SEM, n = 3.

**Figure 4:**
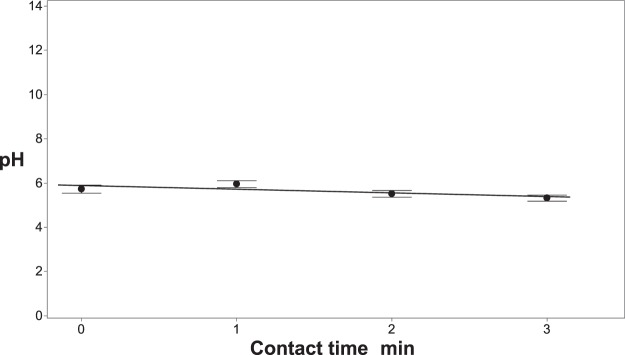
The pH of the solution during treatment as a function of contact time, while maintaining a constant current density of 0.76 mA/cm^2^. The test solution only contained deionized water with free floating mycelia and did not contain fertilizer or chloride salts for the experiment. Error bars are ±SEM, N = 3.

Figure 3 shows the inactivation of *R*. *solani* when applying a current density of 0.76 mA/cm^2^ without the presence of fertilizer and chloride. When the soluble fertilizer and chloride are absent and a low current density (0.76 mA/cm^2^) is applied there is a detectable inactivation of *R*. *solani*. Pathogen inactivation increased with increasing contact time (Fig. 3), with a 77% reduction after a 3-minute exposure. The pH of the solution also remained stable at ~5.5 throughout all contact times that were tested (Fig. 4).

The observed inactivation under these conditions may be due to the acidic environment in the immediate vicinity of the anodes^30^. At low pH levels non-enzymatic proteolysis can occur on the mycelial sheath of *R*. *solani*, ultimately leading to cell death if the exposure is sufficient^42–44^. Structural changes to microorganisms, such as the cleavage of proteins, have been shown to occur at a constant potential of 0.8 and 1.0 V^44,45^ but higher potentials are likely needed to cleave polysaccharides. The operating voltage for this specific experiment was ∼32 V, which could be sufficient for this process to occur. This higher applied potential could also induce electroporation, a process by which the cell membrane becomes increasingly permeable, ultimately leading to loss of function and cell death^46–51^. Increasing the porosity of the cell envelope leads to leakage of cellular material^38^. However, this effect has also been shown to be “species-specific” in studies with gram-negative and gram-positive bacteria^46,51^. Other possible mechanisms include direct oxidation of polysaccharides at the anode^52^, protein extraction (cleavage of disulfide and/or peptide bonds due to the discharge of water and dissolved oxygen molecules) at the cathode^53^, or reductive hydrogen peroxide production at the cathode^30^. It is difficult to isolate a specific mechanism of the observed inactivation (Fig. 3) and in all likelihood, it is a combination of processes leading to the partial inactivation observed^54,55^.

#### Inactivation of *R. solani* in a solution containing chloride

**Figure 5:**
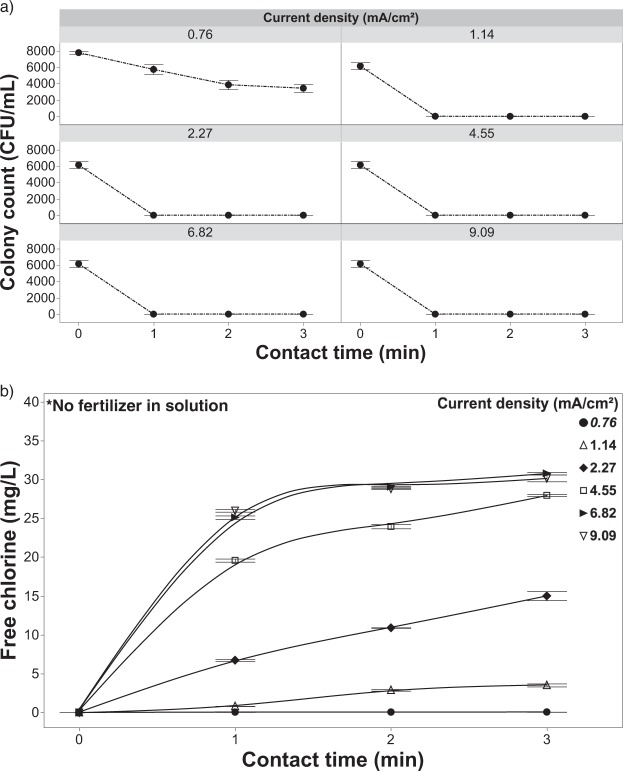
Inactivation and free chlorine dynamics in the absence of fertilizer. (**a**) Inactivation of *R. solani* as a function of current density and contact time with 20 mg/L of chloride in solution with no fertilizer present, and (**b**) effluent free chlorine concentration from the EFC after treatment of *R. solani* with 20 mg/L of chloride with no fertilizer present. Error bars are ±SEM, N = 3.

In the absence of fertilizer salts, the EFC achieved complete pathogen inactivation at all but the lowest current densities (0.76 & 1.14 mA/cm^2^) for the 1-min contact time when chloride (20 mg/L) was present in the solution (Fig. 5a). The 20 mg/L chloride concentration was chosen as it was well below the reported phytotoxic thresholds of most crops (species specific)^24^ and showed efficacy in preliminary testing (data not shown). At a current density of 0.76 mA/cm^2^ pathogen inactivation was greatly enhanced in comparison to the previous experiment in which there was no chloride or fertilizer present (Figs 3 and 5a). Although, when considered in combination with the results shown in Fig. 5, it appears that a current density of 0.76 mA/cm^2^ still does not provide the required energy to generate a sufficient amount of free chlorine to effectively control pathogens. A free chlorine residual was measured and complete inactivation was achieved at a current density of 1.14 mA/cm^2^ after a 2-min contact time, indicating that the critical current density threshold lies somewhere between 0.76 and 1.14 mA/cm^2^. This result shows the EFC’s ability to achieve complete inactivation with a lower contact time of 2-minutes and releasing an even lower free chlorine concentration (∼2.88 mg/L) in direct comparison to the study conducted by Cayanan *et al*., 2009. Beyond a current density of 1.14 mA/cm^2^, complete inactivation was achieved by the 1-min contact time (Fig. 5a), which is expected given the relatively high free chlorine levels achieved at these current densities (Fig. 5b).

**Figure 6:**
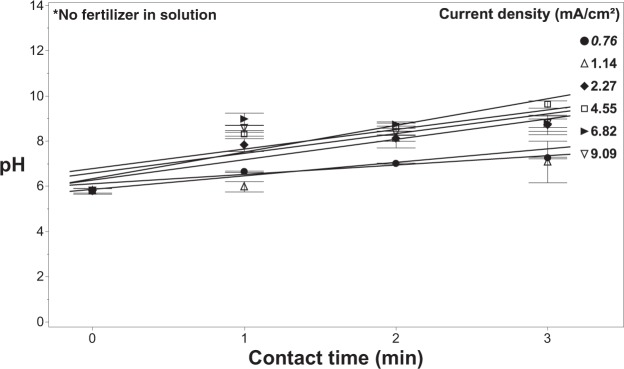
The pH of the solution during treatment as a function of current density and contact time with 20 mg/L of chloride in solution with no fertilizer present. Error bars are ±SEM, N = 3.

Regression analysis was used to characterize the production of free chlorine as a function of contact time and current density. Results indicate that there is a significant relationship (R^2^ = 0.60; p < 0.0001) between contact time and current density. The pH was shown to respond proportionally to contact time, with pH increasing from 5.5 to as high as 8.5 at the longest contact times (Fig. 6).

The free chlorine residuals observed in this experiment (Fig. 5b) are well beyond reported phytotoxic thresholds with a residual of ∼25 mg/L for 6.82 mA/cm^2^ at the 1-minute contact time^6,19,24,56^. Under these solution conditions (e.g., raw irrigation water with trace chloride levels) there would be a need to include a free chlorine stripping step prior to crop application^19,20,23^. This said, under these conditions the system could be used to generate sufficient free chlorine residuals to provide system-wide disinfection between crop cycles.

### Free chlorine evolution with increasing fertilizer and current density

**Figure 7:**
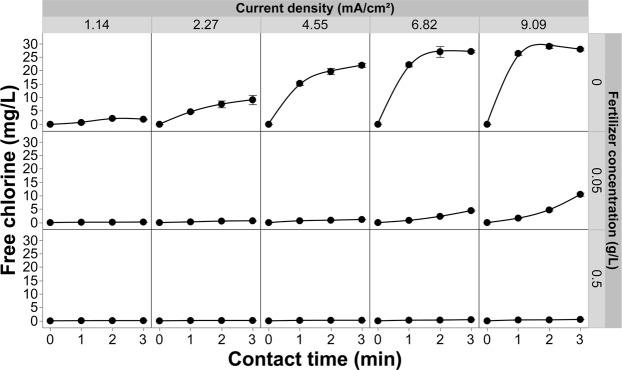
Effluent free chlorine measured from the EFC as a function of current density, contact time, as well as varying the concentration of fertilizer in solution. All of these experiments were conducted with a chloride concentration of 20 mg/L. Error bars are ±SEM, N = 3. Where error bars are not shown they are too small to be visible behind the symbol.

In most greenhouse crop production systems, fertilizer is delivered via the irrigation solution [fertigation], so it was critical to evaluate the pathogen inactivation efficacy in the presence of a representative commercial fertilizer. Effluent free chlorine levels at three different concentrations of commercial fertilizer were evaluated at a fixed chloride concentration of 20 mg/L in the absence of *R*. *solani* (Fig. 7). This chloride concentration is compatible with crop production^24^ but still provides sufficient chloride to generate phytotoxic levels of free chlorine in the absence of a free chlorine stripping step (Fig. 5b)^19^. Figure 7 shows a positive correlation between the production of free chlorine and the contact time of the solution with the electrodes. Further, when increasing the current density there is a large increase in the amount of free chlorine produced in bulk solution when fertilizer levels are low (0 and 0.05 g/L). Although the free chlorine levels rose quickly they reach a plateau at about the 2-min contact for current densities of 6.82 and 9.09 mA/cm^2^ when fertilizer was not present. The data presented in Fig. 5 indicates that the concentration of effluent chlorine at lower current densities is current limited, while at higher current densities the system becomes mass transport limited^57^. Mass transport limitation was due to depletion of chloride ions at the anode-solution interface as local supplies of chloride were transformed to free chlorine. At higher current densities the conversion of chloride to free chlorine was greater than the replenishment rate of chloride, which lead to the observed plateau (Figs 5b and 7). When fertilizer was introduced into the test solution, even in small amounts (i.e., 0.05 g/L), the free chlorine residuals decreased by as much as two orders of magnitude (Fig. 7, middle row panels). At the manufacturer’s recommended application rate of 0.5 g/L, the free chlorine dropped to <1 mg/L; well below reported phytotoxic thresholds (~2.5 mg/L).

The introduction of fertilizer clearly reduced the residual free chlorine concentrations in the test solutions (Fig. 7). Given the significant drop in free chlorine levels, even at modest fertilizer concentrations, it is important to consider the fertility regime when determining contact times and current densities in these types of systems. The dramatic reduction in effluent free chlorine in the presence of fertilizer indicated that there was a competing sink for free chlorine. If the competing sink is more effective at consuming free chlorine than the pathogen inactivation mechanisms then the system would not be effective for pathogen control in typical greenhouse production systems. Conversely, if the pathogen inactivation mechanisms predominate then it may be possible to control pathogens while taking advantage of the secondary sink present in the fertilizer to reduce the free chlorine levels below reported phytotoxic thresholds.

**Figure 8:**
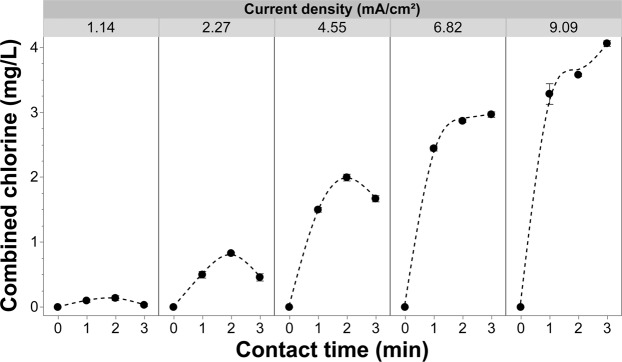
Effluent combined chlorine measured from the EFC as a function of current density and contact time, while maintaining the concentration of fertilizer at 0.5 g/L. All of these experiments were conducted with a chloride concentration of 20 mg/L. Error bars are ±SEM, N = 3.

One competing sink considered was the formation of chloramine. Several of the test solutions contained NH_4_^+^, which can react with free chlorine [break point chlorination] to generate chloramine species (Fig. 8). Although data on chloramine phytotoxicity is surprisingly limited, there is some evidence that the overall phytotoxicity of these chlorinated species is lower than that reported for free chlorine, at least in susceptible crops such as lettuce^25,26^. It may simply be that chloramine toxicity is a function of its greater stability in solution and therefor the contact times or potential exposure is much higher for these chemical species than it is for free chlorine species such as hypochlorous acid. In the presented study, chloramine production did exceed that reported to be phytotoxic in hydroponically grown lettuce^25^. Additional crop production studies are needed to clarify the issue of chloramine phytotoxicity across a range of crops and production systems. Until that data is available, some caution should be exercised when using any chlorination system, including the one presented here^25,26^. This said, the chloramine phytotoxicity threshold in media-based systems does not appear to be as low as in strict hydroponic culture^58^.

**Figure 9:**
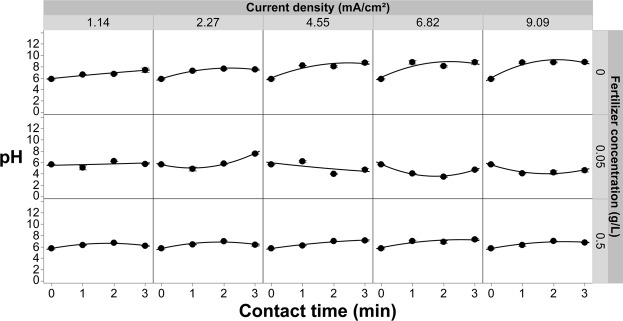
Effluent pH measured from the EFC as a function of current density, contact time, as well as varying the concentration of fertilizer in solution. All of these experiments were conducted with a chloride concentration of 20 mg/L. Error bars are ±SEM, N = 3.

**Figure 10:**
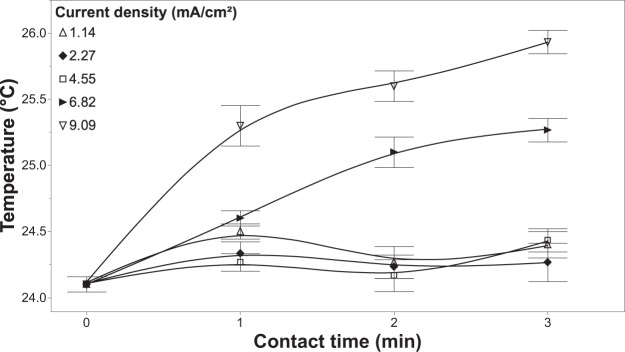
Temperature measurements from the EFC as a function of current density and contact time at a fertilizer concentration of 0.5 g/L and 20 mg/L of chloride in solution. Error bars are ±SEM, N = 3.

There are other factors that also were needed to determine the electrochemical dynamics during treatment, such as changes in pH and temperature. The pH of the solution was shown to remain in the neutral range with the introduction of fertilizer, while the absence of fertilizers is shown to increase in pH with longer contact times (Fig. 9). Furthermore, the temperature of the solution was shown to increase as high as 1.5 °C by using higher current densities (Fig. 10). Although, using lower current densities only changes the temperature of the solution by a maximum of 0.5 °C. Although there was some variation in pH and temperature, these factors should not affect the performance of the system in treating fertigation solutions and on plant growth.

### *Rhizoctonia solani* inactivation experiments

#### Pathogen inactivation in the presence of fertilizer

In order to determine the influence of the competing free chlorine sinks on pathogen inactivation efficacy, it was important to determine *R*. *solani* inactivation in the presence of fertilizer while increasing both chloride concentrations and current densities.

**Figure 11:**
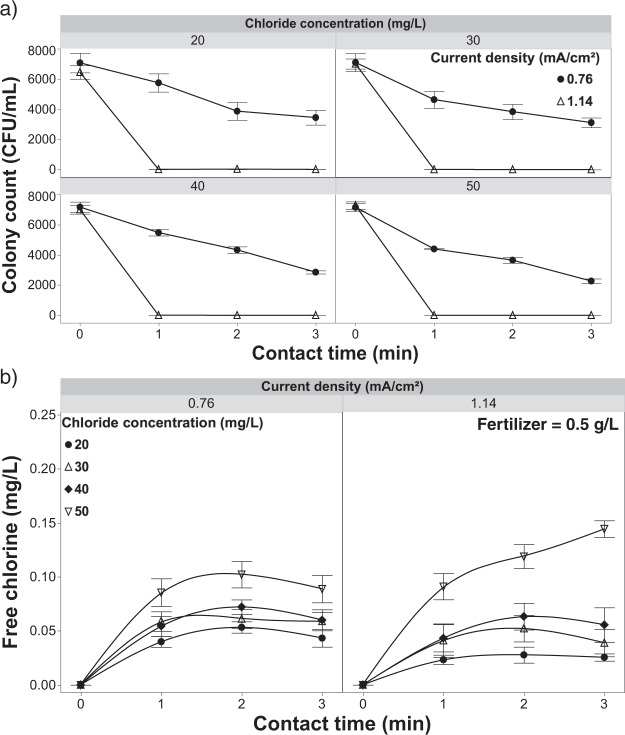
*Rhizoctonia solani* inactivation and free chlorine dynamics with increasing chloride concentration. (**a**) Inactivation of *R. solani* as a function of current density with a fertilizer concentration of 0.5 g/L and increasing chloride concentration, and (**b**) effluent free chlorine concentrations observed under the same parameters while increasing the concentration of chloride. Error bars are ±SEM, N = 3.

**Figure 12:**
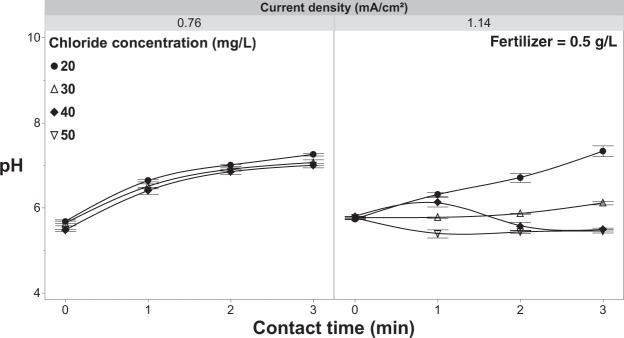
The pH of the solution as a function of current density with a fertilizer concentration of 0.5 g/L and increasing chloride concentration. Error bars are ±SEM, N = 3.

When chloride concentrations were increased from 20 to 50 mg/L under the lowest current density (0.76 mA/cm^2^), there was a modest increase (17%) in pathogen inactivation but not sufficient to result in complete inactivation even at the 3-min contact time (Fig. 11a). Increasing the current density to 1.14 mA/cm^2^ resulted in complete inactivation at chloride levels 30, 40 & 50 mg/L beyond a 2-min contact time (Fig. 11a). Once again, at both current densities, the free chlorine residuals in the outflow were well below phototoxic thresholds (Fig. 11b). When increasing the concentration of reactants (chloride) at the anode surface, free chlorine concentrations in the bulk solution will increase concomitantly due to mass transport mechanisms that move the free chlorine away from the anode and into the bulk solution^57,59^. Increased bulk solution free chlorine concentrations likely lead to the increased inactivation rates as more pathogen propagules would come into contact with free chlorine in the bulk solution relative to the limited anode surface area. Furthermore, forming more HOCl in bulk solution is also reflected with a decreasing pH by increasing the current density and chloride concentration (Fig. 12).

**Figure 13:**
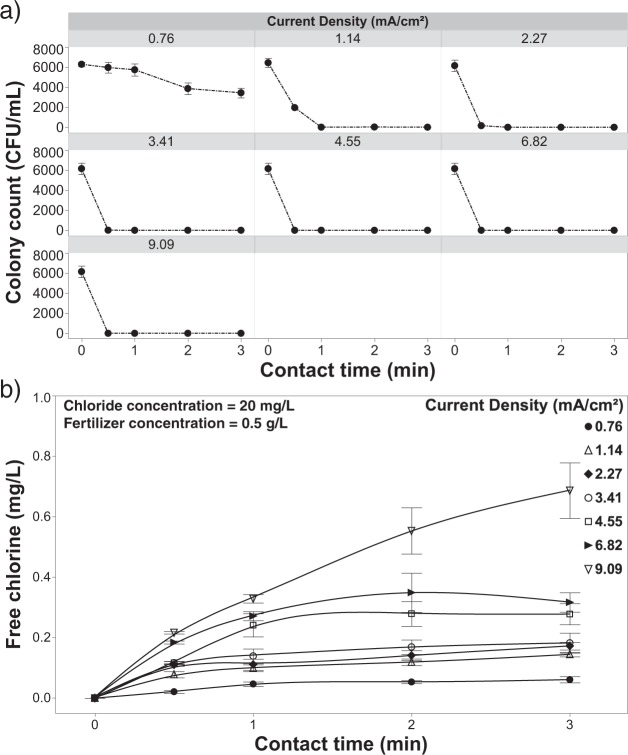
*Rhizoctonia solani* inactivation and free chlorine dynamics with increasing current density. (**a**) *R. solani* inactivation as a function of current density and contact time with 20 mg/L of chloride and 0.5 g/L of fertilizer in solution and (**b**) effluent free chlorine from the EFC with applying variable current densities and contact times with the same test solution. Error bars are ±SEM, N = 3.

Figures 11a and 13a highlight the need for further study at shorter contact times (<30 seconds) than were achievable with the presented set-up. Achieving better resolution for pathogen inactivation at shorter contact times/higher flow rates, various sub-phytotoxic chloride concentrations, and compatible current densities would further inform the engineering requirements for larger scale systems.

Free chlorine generation was monitored while maintaining constant current densities (0.76 mA/cm^2^ and 1.14 mA/cm^2^) over increasing chloride concentrations (20 mg/L to 50 mg/L) (Fig. 11b). In this scenario there was a marginal increase in free chlorine leaving the EFC (Fig. 11b). However, this increase in free chlorine generation was minimal given the 2.5-fold increase in chloride concentration (Fig. 11). It is clear that under the conditions tested, the manipulation of other process control mechanisms (e.g., current density and contact time) are more influential in achieving pathogen inactivation. Due to the complexity of the solution matrix, more research is needed to expand on inactivation rates, free chlorine production, and effluent free chlorine models in order to discover the thresholds for the proposed inactivation mechanisms (Figs 3–11). This should be conducted in relation to current and mass transport limitations to better understand primary, secondary and tertiary mechanisms for inactivation in the presence of fertilizer.

#### Inactivation of *R. solani* as a function of current density

Increasing the current density while maintaining a constant chloride concentration (20 mg/L) increased pathogen inactivation efficacy (Fig. 13a). As current density increased more electrons became available [at the anode surface] to participate in the reactions leading to the formation of free chlorine. As this charge transfer increased, the amount of free chlorine produced was no longer current limited; rather, it became governed by the mass transfer efficiency of the system (Figs 5b and 7)^57,59,60^. The increased free chlorine concentration at the anode supports a greater flux to the bulk solution where it is available to further react with free-floating pathogen cells. The EFC achieved higher inactivation rates of *R*. *solani* after a 1-min contact time, with the exception of the lowest current density (0.76 mA/cm^2^) (Figs 5a and 11a, 13a). When increasing the current density to 1.14 mA/cm^2^, there was a 2.80 log reduction for the 1-minute contact time and beyond. When increasing the current to 2.27 or 3.41 mA/cm^2^, a 3.75 log reduction was achieved at the 1-minute contact time. A current density of 4.55 mA/cm^2^ achieved similar log reductions to lower current densities but inactivation was achieved in half the time (30 seconds). The highest current densities (6.82 and 9.09 mA/cm^2^) resulted in complete inactivation at all contact times tested. Further testing at these higher current densities and shorter contact times <30 s should be conducted to further characterize the system and inform the development of larger scale systems.

**Figure 14:**
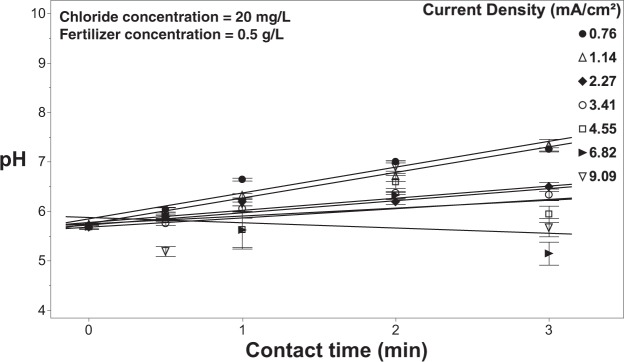
The pH of the solution as a function of current density and contact time with 20 mg/L of chloride and 0.5 g/L of fertilizer in solution and (**b**) effluent free chlorine from the EFC with applying variable current densities and contact times with the same test solution. Error bars are ±SEM, N = 3.

When fertilizer is present in the test solution the effluent free chlorine residuals were consistently and considerably lower (<0.8 mg/L free chlorine) (Fig. 13b) than solutions that did not contain fertilizer (Fig. 5b) regardless of the current density applied. One of the main concerns with using free chlorine to treat irrigation water is phytotoxicity, which can occur at effluent concentrations as low at ~2.5 mg/L^19,24^. In this study, the addition of fertilizer salts, at concentrations consistent with commercial production, reduced effluent free chlorine to levels compatible with crop production^19,24^; yet, the addition had negligible impacts on pathogen inactivation efficacy. Even at the highest current densities and contact times achieved with the current system, the effluent free chlorine residuals still remained well below reported phytotoxic thresholds for free chlorine (Fig. 7). The ability to apply these high currents and/or long contact times, without the threat of exposing the crop to phytotoxic levels of free chlorine, suggests that even recalcitrant pathogens could be controlled through the manipulation of contact time and/or current density. However, as noted earlier, chloramine formation and its associated phytotoxicity needs to be considered, especially at higher current densities (>1.14 mA/cm^2^)^25,61^. Although a concern, the production system used (e.g., solution culture versus media-based) and the specific sensitivity of the crops grown will heavily influence the overall phytotoxicity. On a system and crop-specific basis higher current densities could likely be used without phytotoxic effects^58,62^. Contact time is also a major contributing factor when considering the phytotoxicity of any disinfectant. When comparing hypochlorous acid to chloramine in terms of phytotoxicity, it may be that the longer contact times achieved with chloramine, as a result of its greater stability, is the major factor in determining phytotoxic thresholds. Further studies aimed at clarifying this contact time effect are needed in order to develop the best possible management practices when employing chlorination for fertigation water disinfection. Lastly, the pH was shown to have an inverse relationship with current density, with the bulk solution gradually becoming more acidic (Fig. 14). Although, the pH was shown to remain relatively constant with using current densities 2.27–4.55 mA/cm^2^.

### Nitrogen dynamics in the EFC system

**Figure 15:**
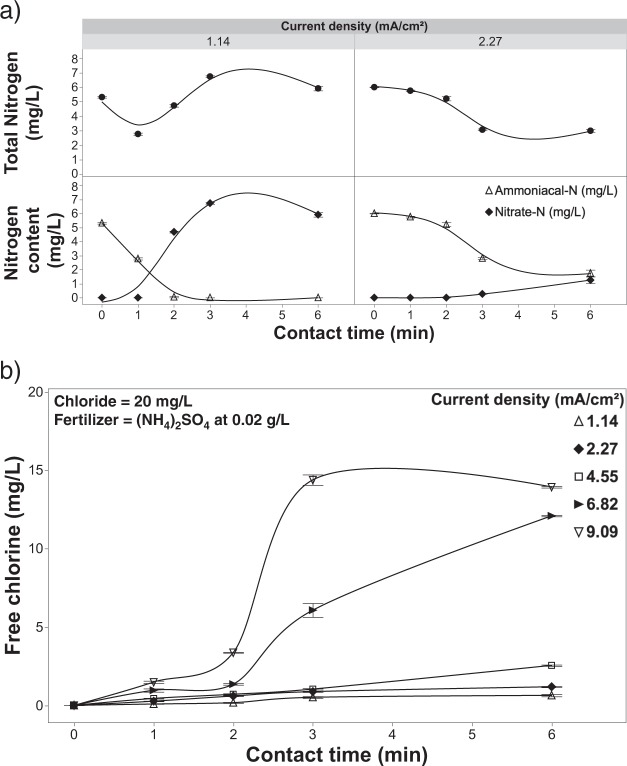
Nitrogen and free chlorine dynamics at variable contact times and current densities. (**a**) Nitrogen dynamics as a function of current density (1.14 & 2.27 mA/cm^2^) and contact time of the solution containing (NH_4_)_2_SO_4_ as the supporting electrolyte (relative to 0.05 g/L of 20-8-20 Plant Prod fertilizer) and 20 mg/L of chloride. (**b**) effluent free chlorine concentration as a function of current density and contact time. Error bars are ±SEM, N = 3.

The reduction in free chlorine residuals in the presence of fertilizer containing ammonium (Fig. 7) suggests that breakpoint chlorination occurred during treatment. Ammonium sulphate was used as a supporting electrolyte at a concentration of 0.02 g/L, which corresponds to the total nitrogen in the 0.05 g/L fertilizer solutions used in the other experiments presented (Fig. 7). At a current density of 1.14 mA/cm^2^, the concentration of ammonium was inversely proportional to the contact time. However, the concentration of nitrate, which was not initially present, was also shown to be proportional to the contact time. The result was no net change in total nitrogen (Fig. 15a). At lower current densities, the conversion favoured nitrification (Fig. 15a). At higher current densities (e.g., 2.27 mA/cm^2^) nitrification also occurred; however, denitrification also became part of the overall process ultimately leading to a small net loss of total nitrogen from the solution^32^. The amount of nitrogen lost from the solution increased modestly with increasing current densities (4.55–9.09 mA/cm^2^; data not shown).

**Figure 16:**
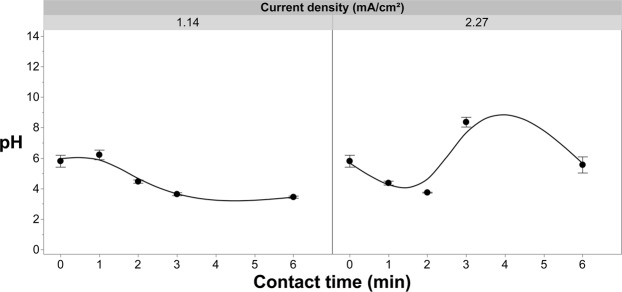
The pH of the solution as a function of current density (1.14 & 2.27 mA/cm^2^) and contact time of the solution containing (NH_4_)_2_SO_4_ as the supporting electrolyte (relative to 0.05 g/L of 20-8-20 Plant Prod fertilizer) and 20 mg/L of chloride. Error bars are ±SEM, N = 3.

Free chlorine was shown to variably increase between the current densities and the contact times applied to the solution when containing ammonium sulphate (Fig. 15b). However, these levels of effluent free chlorine are smaller than the amount leaving the EFC in bulk solution when no fertilizer was present (Fig. 5b). The suspected cause for this decreased effluent free chlorine with the presence of fertilizer is due to the consumption by ammonium and transformations to other nitrogenous species due to direct oxidation by the anode and indirect oxidation from free chlorine^63–66^. Chlorine is known to react with various organic and inorganic components in water, such as microorganisms but also including ammonium and ferrous iron^1,37,67^. The reaction rates between the organic and inorganic fractions can vary considerably but are largely determined by stoichiometric kinetics and the pH of the solution^37,67^. Breakpoint chlorination of ammonium/ammonia is highly pH sensitive with the highest reaction rate occurring around a pH of 8.5^32^. Maintaining the pH between 5.5 and 6.5, which is compatible with crop requirements, limits the amount of nitrogen lost from the system (Fig. 16). The amount of nitrogen lost will be limited, though the formation of chloramines can still occur at these lower pH levels. The surface of the anodic working electrode for RuO_2_ produces portions of N_2_, NO_2_^−^ and NO_3_^−^, which are increasingly formed with higher pH and current densities^68^. The conversion rates of these products are limited in undivided electrochemical cells, such as the one presented herein, in comparison to divided cells^68^. Thus, the loss of total nitrogen will be limited due to the characteristics of the fertigation solution being treated and the use of an undivided cell.

At higher current densities, breakpoint chlorination and the formation of chloramines has been demonstrated as the most likely mechanism for the consumption of free chlorine, while lower current densities favoured the formation of secondary reactions such as nitrate generation^32,37,67,69,70^. Although breakpoint chlorination is likely the predominant secondary sink for free chlorine in the presented process, the consumption of excess (beyond pathogen inactivation demand) free chlorine cannot be solely relied upon as only being caused by breakpoint chlorination. Previous literature has shown that other components in fertilizer also contribute to the consumption of free chlorine, such as Ethylenediaminetetraacetic acid (EDTA), Fe^2+^ and Mn^2+^ ^71–73^. Furthermore, electrochlorination with Ti/RuO_2_ and stainless-steel electrodes has been demonstrated for the degradation of EDTA and decomplexation of chelated metals^74,75^. However, the complete degradation of EDTA was only achieved when using a current density of 7.52 mA/cm^2^, a pH of 9, chloride concentration of 3000 mg/L, and a contact time of approximately 250 minutes.

### Nutrient stability

Individual macronutrient ions from the 0.5 g/L fertilizer solution treated at a current density of 4.55 mA/cm^2^ were measured. This current density was more than sufficient to inactivate pathogens under all test conditions, yet maintained residual free chlorine levels below phytotoxic levels in the presence of fertilizer (Figs 4a and 5, 8a). The effluent concentration of chloride was shown to decrease in proportion to the amount of free chlorine leaving the EFC, which is consistent with the expected chlorine mass balance. Less transient chloride reductions could be due to degassing (Cl_2(g)_), chloramination or free chlorine reacting inside pathogen cells and becoming bound^37,66^. Nonetheless, the concentration of chloride in the effluent was found to be stable, demonstrating that *in situ* electrochemical hypochlorination will continue to regenerate free chlorine while conserving chloride. All other measured nutrients were stable under the test conditions (data not shown) as determined by slope analysis (all slopes = 0 at p < 0.05), which demonstrated that nutrients can be conserved in this system under prescribed operational conditions.

Higher current densities and longer contact times were also tested and found to have only minor effects on select nutrient ions (NO_3_^−^, K^+^ & SO_4_^2−^) (data not shown). It should also be noted that if the pH in the solution is not regulated and is allowed to increase nitrogen could be lost from the fertigation solution and chloramine formation may occur. This said, pH is routinely adjusted in production systems and at typical fertigation solution pH levels (i.e., 5.4–6.5) nitrogen should be stable. Although only minor effects on macronutrient ions were noted in this study, other fertilizer types should be examined to ensure general compatibility across fertilizers types and manufacturers. It should be noted that micronutrients (e.g., iron, manganese, etc.) were not examined in this study. The influence of regenerative *in situ* hypochlorination on micronutrient dynamics needs to examined prior to any use in a commercial setting.

### Direct operational costs

The electrolytic power cost was considered for the 2.27 mA/cm^2^ current density profile (i.e., costs of ancillary equipment not included in calculations). This current density resulted in a high pathogen inactivation rate within a 1-min contact time or a flow rate of 380 mL/min (Fig. 8a), which is sufficient to accommodate a bench-scale crop production trial with a fertigation reservoir of 200 L. Under continuous operation, the current EFC configuration would use 0.447 kWh for treating 1000 L of solution. At an energy price of $0.12/kWh this translates to a treatment cost of $0.05/m^3^. However, the time needed for treating this volume of irrigation solution is 43.86 hours. If using a current density of 4.55 mA/cm^2^ at nearly double the flow rate (720 mL/min), the cost remains the same but the treatment time is reduced to 23.15 hours. These calculations are basic and do not account for additional energy and hardware costs, nor do they represent a reasonable treatment cycle. What these examples do show is the flexibility of the process and its potential upon scaling. Disinfection targets can be achieved through the manipulation of several key parameters (current density, flow rate/contact time, chloride concentration, electrode area (scaling factor)), which provides the operator with multiple options when addressing specific treatment challenges.

### Conclusion

The regenerative *in situ* electrochemical hypochlorination system described has been demonstrated to effectively inactivate *R*. *solani* in fertigation test solutions under laboratory conditions. Pathogen control can be achieved through the manipulation, in isolation or in combination, of the applied current, contact time, and the concentration of chloride in solution. Applying a low current density and increasing the concentration of chloride can moderately increase pathogen inactivation rates. Maintaining a low concentration of chloride (20 mg/L) and adjusting the current density and/or the residence time (flow rate) in relation to the free chlorine demand proved to be effective for pathogen inactivation. The effluent free chlorine concentration, when fertilizer was present, was below the phytotoxic threshold (~2.5 mg/L) reported by others; however, some caution should be exercised due to the formation of chloramines that can be phytotoxic to some crops at lower concentrations than hypochlorous acid [free chlorine], particularly in solution culture. Chloride concentrations were also shown to remain stable throughout the treatment process. This is in direct contrast to continuous free chlorine dosing, which can lead to the accumulation of chloride, potentially to phytotoxic levels, in recirculating systems. The system does not alter the macronutrient composition under the conditions examined; however, micronutrients (e.g., iron) need to be evaluated. Effective pathogen inactivation combined with sub-phytotoxic free chlorine residuals make the system compatible with greenhouse crop production. Further studies are required to evaluate the efficacy of the system across a range of pathogen types, evaluate scalability and other potential engineering challenges such as the integration with nutrient dosing systems. This study provides the initial baseline data to support further investigations of RuO_2_ DSAs as a regenerative *in situ* hypochlorination system for the control of *Rhizoctonia solani* in commercial greenhouse production.
